# Antimicrobial susceptibility of *Streptococcus pneumoniae* in adult patients with pneumococcal pneumonia in an urban hospital in Mozambique

**DOI:** 10.1186/1756-0500-7-110

**Published:** 2014-02-25

**Authors:** Jeannet C Bos, Sara J Beishuizen, Geoffrey C Madeira, Elmano dos Santos Gomonda, Esmeralda O Cossa, Augusto C Macome, Reindert P van Steenwijk, Constance Schultsz, Jan M Prins

**Affiliations:** 1 Department of Internal Medicine, Division of Infectious Diseases, Academic Medical Centre (AMC), University of Amsterdam, Room F4-217, Meibergdreef 9, Amsterdam, 1105 AZ, The Netherlands; 2 University of Amsterdam (UvA), Faculty of Medicine, Meibergdreef 9, 1105 AZ, Amsterdam, The Netherlands; 3 Department of Medical Microbiology, Academic Medical Centre (AMC), University of Amsterdam, Amsterdam, The Netherlands; 4 Department of Pulmonology, Academic Medical Centre (AMC), University of Amsterdam, Amsterdam, The Netherlands; 5 Amsterdam Institute for Global Health and Development (AIGHD), Trinity Building C, Pietersbergweg 17, Amsterdam, 1105 BM, The Netherlands; 6 Faculdade de Ciências de Saúde, Universidade Católica de Moçambique (UCM), Caixa Postal 821, Beira, Mozambique; 7 Hospital Central da Beira (HCB), Caixa Postal 1613, Beira, Mozambique

**Keywords:** *Streptococcus pneumoniae*, Penicillin resistance, Antimicrobial susceptibility, Pneumonia, Adults, Sub-Saharan Africa

## Abstract

**Background:**

*Streptococcus pneumoniae* is the leading cause of community–acquired pneumonia in Africa. Antimicrobial resistance of *S. pneumoniae* to penicillin and other commonly used antibiotics has increased worldwide. However, prevalence data from the African region are sparse, especially with regard to adults.

**Findings:**

In this study, adult patients presenting at an urban referral hospital in central Mozambique were screened for pneumococcal pneumonia during an 8-week period in 2010: Patients with a respiratory syndrome underwent chest radiography and a sputum sample was collected for pneumococcal culture and antimicrobial susceptibility testing. A urine sample was tested for the presence of pneumococcal antigen.

177 patients with a respiratory syndrome were included. Overall, 41/177 (23%) patients fulfilled criteria for definite or probable pneumococcal pneumonia and in the group of patients with a positive chest x-ray this concerned 35/86 (41%) patients. 166 sputum cultures yielded 16 pneumococcal strains. One mg oxacillin disc testing identified potential penicillin resistance in 7/16 (44%) strains. Penicillin minimal inhibitory concentrations (MICs) were measured for 15 of these strains and ranged from <0.016-0.75 mg/L. No MICs >2 mg/L were found, but 3/15 (20%) pneumococcal strains had MICs >0.5 mg/L. All pneumococci were sensitive to erythromycin as measured by disc diffusion testing, whereas 44% was resistant to trimethoprim-sulfametoxazole.

**Conclusions:**

The proportion of pneumonia cases attributable to pneumococcus appeared to be high. Whilst none of the *S. pneumoniae* strains tested were penicillin resistant, standard penicillin dosing for pneumonia may be insufficient given the observed range of pneumococcal penicillin MICs.

## Findings

### Background

*Streptococcus pneumoniae* is the leading cause of community-acquired pneumonia around the world and in Africa [1]. Severe pneumococcal disease, for which HIV is a major risk factor, is associated with a high mortality in African adults [2,3].

Antimicrobial resistance of *S. pneumoniae* against the most commonly used antimicrobial drugs is increasing worldwide, principally affecting β-lactam, macrolide and sulfonamide sensitivity [4]. In Africa, β-lactam antibiotics, and penicillin in particular, are amongst the most widely used antimicrobial drugs for empirical treatment of pneumonia. However, African antimicrobial resistance data are scarce, especially when concerning adults [5,6].

We investigated the prevalence of penicillin resistance of *S. pneumoniae* in adults with pneumococcal pneumonia presenting in an urban hospital in central Mozambique.

### Patients and methods

#### Study area

Our study was conducted at the Beira Central Hospital (*Hospital Central da Beira*: HCB), a 932-bed governmental referral health facility with 260 internal medicine beds, located in the coastal capital of the central Sofala province of the sub-Saharan African Mozambique. The HIV prevalence among adult women in this region was found to be 22.9% in 2009 [7]. Mozambique also ranks amongst the countries with the highest tuberculosis burden, with an estimated 409 new cases per 100,000 pop/year. Pneumococcal vaccination of infants as part of the Mozambican National Immunization Programme only started in April 2013.

#### Study design

We carried out a cross-sectional study among adults (≥ 16 years) presenting at the HCB emergency room with a respiratory syndrome. A respiratory syndrome was defined as fever (axillary body temperature ≥37.5°C) in combination with one or more of the following complaints: cough with or without sputum production, dyspnoea and chest pain. Study participants were recruited prospectively during a study period of 8 weeks, from April to June 2010. A posterior-anterior chest x-ray (CXR) was made immediately after presentation and subsequently digitalized for review by an experienced, external pulmonologist, who was blinded to the study participant’s clinical data and pneumococcal test results.

Written informed consent was obtained from each patient before study entry. Ethical approval was obtained from the Mozambican National Committee of Bio-ethics, through its sub-committee seated in Beira (ref.: 007/10/SBCE).

#### Study procedures

Sputum samples were collected from all patients for pneumococcal culture and sensitivity testing. Patients were carefully instructed about how to provide a good-quality sputum sample in a standardized way and study staff directly observed the sputum production process for all study participants. Sputum was inoculated on selective Columbia CNA sheep blood agar plates (bioMérieux SA, Marcy l’Etoile, France). Plates were incubated overnight at 35–37°C in a candle jar after placement of a 5 μg optochin disc (Oxoid Ltd., Cambridge, UK). Colonies suspected for pneumococcus, on the basis of colony morphology and optochin susceptibility as per manufacturer’s instructions, were subcultured on sheep blood agar plates (bioMérieux SA, Marcy l’Etoile, France). The Dryspot Pneumo latex agglutination test (Oxoid Ltd., Cambridge, UK) was used as per manufacturer’s instructions for final identification.

Antimicrobial susceptibility of pneumococcal isolates against oxacillin (1 μg), trimethoprim-sulfamethoxazole (co-trimoxazole: 5.2-240 μg) and erythromycin (78 μg) was tested using disc diffusion method (Neo-Sensitabs, Rosco Diagnostica, Taastrup, Denmark). Zone diameter breakpoint interpretation was done according to European Committee on Antimicrobial Susceptibility Testing (EUCAST) recommendations as recommended by the manufacturer. The benzyl penicillin minimal inhibitory concentration (MIC) was measured using E-test strips (bioMérieux SA, Marcy l’Etoile, France). Clinical breakpoint interpretation of these MICs was done using the EUCAST clinical breakpoint tables for interpretation of MICs, using the recommendations for non-meningeal infections: MIC ≤ 0.06 mg/L: susceptible; > 2 mg/L: resistant; > 0.06 and ≤ 2 mg/L: intermediate category with dose specific breakpoints [8].

A urine sample was collected from all patients for urinary pneumococcal antigen testing using Binax NOW *S. pneumoniae*, according to the manufacturer’s instructions (Binax Inc., Portland, ME, USA; currently manufactured by Alere Inc., Waltham, MA, USA).

#### Data analysis

**Figure 1:**
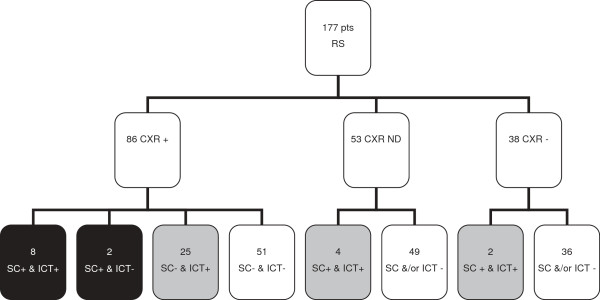
**Study profile.** Pts: patients; RS: respiratory syndrome; CXR: chest x-ray; ICT: immunochromatographic test (urine pneumococcal antigen test); SC: pneumococcal sputum culture; +: positive; -: negative; ND: not done; Colour/pattern boxes: black: definite pneumococcal pneumonia; grey: probable pneumococcal pneumonia.

In the absence of a ‘gold standard’ definition of (suspected, pneumococcal) pneumonia and given the lack of state-of-the-art blood culture capacity at the study site, the following definitions were used. Suspected pneumonia was defined as a respiratory syndrome combined with a positive CXR, as defined by showing evidence of any consolidation, and/or pleural effusion, and/or interstitial disease. Definite pneumococcal pneumonia was defined as a respiratory syndrome in combination with a positive CXR and a positive sputum culture with *S. pneumoniae*. Probable pneumococcal pneumonia was defined as a respiratory syndrome in combination with a positive CXR and a positive ICT. In the absence of a CXR, or whenever a CXR was not positive, a respiratory syndrome with a positive sputum culture with *S. pneumoniae* AND a positive ICT was also defined as a probable pneumococcal pneumonia (Figure 1).

Data were entered and analysed using the Epi Info package version 2002 (CDC, Atlanta, GA).

### Results

#### Study population

177 patients were screened. 97/177 (55%) patients were female and 150/177 (85%) patients were between 16–45 years. HIV status was known for 105/177 (59%) and in this group 84/105 (80%) patients were HIV positive. When asked about antibiotic use in the previous three months, 51/177 (29%) of patients was not able to answer this question because they were uninformed, physically unable to answer or because of a combination of the two. 48/177 (27%) of patients confirmed the use of antibiotics and another 78/177 (44%) denied its use. 30/177 (17%) of patients were receiving antibiotics at the time of study inclusion. Although the name ‘penicillin’ was often mentioned in this group (70%), it was not possible to verify this information in most cases. 13/177 (7%) of patients were found to be on TB treatment at study inclusion.

#### Chest x-rays, culture results and urinary antigen testing

53/170 (31%) patients had a positive urinary antigen test. In 124 patients a CXR was done. 86/124 (69%) patients had a positive CXR and were therefore qualified as having suspected pneumonia. 35 of these 86 (41%) patients turned out to have pneumococcal pneumonia, based on a positive culture and/or a positive urinary antigen test. An additional 6 patients had a positive culture and a positive urinary antigen test in the absence of a positive CXR, so overall, 41/177 (23%) patients with a respiratory syndrome fulfilled the criteria of either definite or probable pneumococcal pneumonia (Figure 1).

A total of 166 sputum samples were collected and subsequent culture yielded 16 pneumococcal strains (Table 1). One mg oxacillin disc testing identified potential penicillin resistance in 7/16 (44%) strains. One pneumococcal strain did not survive for further testing and as a result, MICs were measured for 15 pneumococcal strains using the E-test. Benzyl penicillin MICs ranged from <0.016 to 0.75 mg/L. No isolates were found to be resistant to penicillin (MIC >2.0 mg/L). However, 3/15 (20%) pneumococcal strains had MICs >0.5 mg/L, and would classify as non-susceptible when a dose of penicillin of 1.2 g (2 million IU) 4 times daily or less is used. Based on disc diffusion testing a 100% susceptibility rate was observed for erythromycin, whereas 7/13 (44%) strains were resistant to co-trimoxazole.

**Table 1:** Pneumococcal antimicrobial susceptibility testing results

**Strain**	**Penicillin MIC (mg/L)**	**Oxacillin zone diameter BI**	**Erythromycin zone diameter BI**	**Co-trimoxazole zone diameter BI**
1	<0.016	S	S	I
2	<0.016	S	S	S
3	0.016	S	S	R
4	0.016	S	S	I
5	0.016	S	S	S
6	0.016	S	S	I
7	0.016	S	S	I
8	0.023	S	S	S
9	0.047	S	S	R
10	0.047	NS	S	I
11	0.125	NS	S	R
12	0.25	NS	S	R
13	0.75	NS	S	R
14	0.75	NS	S	R
15	0.75	NS	S	R
16	ND	NS	S	I

Minimal inhibitory concentration (MIC) and zone diameter breakpoint interpretation (BI) of cultured pneumococcal strains, as measured by E-test (penicillin) and disc diffusion method (oxacillin, erythromycin, co-trimoxazole), respectively. S: susceptible; NS: non-susceptible; R: resistant. In pneumonia, when a dose of 1.2 g (2 million IU) × 4 is used, isolates with MIC ≤0.5 mg/L should be regarded as susceptible to benzyl penicillin; with a dose of 2.4 g × 4 or 1.3 g × 6, isolates with MIC ≤1 mg/L should be regarded as susceptible; and with a dose of 2.4 g × 6, isolates with MIC ≤2 mg/L should be regarded as susceptible [8]. Not done: ND.

### Discussion

Our study presents the first data concerning susceptibility to commonly used antibiotics in adult pneumococcal pneumonia in urban Mozambique. As expected, our study population mainly consisted of young adults with an estimated high HIV prevalence. With 41% of probable pneumonia patients diagnosed with pneumococcal pneumonia, the proportion of pneumonia attributable to pneumococcus was apparently high. All cultured pneumococcal strains were found to be penicillin susceptible, but 3/15 strains had penicillin MICs > 0.5 mg/L, for which dose specific clinical breakpoints apply, according to the EUCAST recommendations.

The estimated high contribution of *S. pneumoniae* to the local burden of pneumonia as found in our study seems to match the results of etiological studies in Africa, where *S. pneumoniae* was the most common causative agent, being found in 31-46% of pneumonia cases [9-11].

After a critical appraisal of microbiological, pharmacokinetic and pharmacodynamic data, as well as clinical studies on the use of penicillin in non-meningeal pneumococcal infection, current penicillin MIC breakpoints for treatment of non-meningeal *S. pneumoniae* infections from the American Clinical and Laboratory Standards Institute (CLSI) have been expanded and re-set at ≤2 mg/L (susceptible) and ≥8 mg/L (resistant) in 2008 [12]. In contrast, for pneumonia, EUCAST categorizes pneumococcal strains with MICs >2 mg/L as resistant while recommending dosage specific MIC breakpoints for strains with MICs ≤ 2 mg/L. EUCAST suggests for each country to apply the breakpoints appropriate for the dosage of benzyl penicillin most often used [8].

As for adult benzyl penicillin treatment in Mozambique, the national formulary recommends the use of ‘1 million International Units (IU) or more, 4–6 times daily’ for non-meningeal infections [13]. MICs as measured in our study do justify the continued use of penicillin in adult pneumococcal pneumonia in our setting. However, current EUCAST’s interpretation of clinical MIC breakpoints supports the use of a standard dosage that would be effective against all strains with penicillin MICs within the observed range, i.e. 2.4 g (4 million IU) 4 times daily or 1.2 g (2 million IU) 6 times daily (Table 1).

Pneumococcal susceptibility rates appear to vary across Africa, but a comparison of data is complicated by the use of different antimicrobial susceptibility testing methods and interpretive criteria. In a recent systematic review on community acquired bloodstream infections in Africa, 9.7% (range 0-36%) of pneumococcal isolates was resistant to ampicillin, 40% to co-trimoxazole and 2% to erythromycin [14]. South Africa, -one of Mozambique’s neighbouring countries-, reported the most impressive pneumococcal resistance rates in the region with 25-50% of isolates being resistant to penicillin (MIC >2 mg/L) [15]. In contrast, in neighbouring Malawi, hospital surveillance data demonstrated that the rate of potential penicillin non-susceptibility based on disc diffusion testing in invasive pneumococcal strains has been approximately 10% since 2005, which appears to be lower than the rate found in our study [16]. However, in this study, MICs were not determined. Co-trimoxazole resistance is common in Africa and our study results are not different in this respect [17].

Our results have to be interpreted with caution as the pneumococcal culture sample size was small and a positive sputum culture may represent colonization rather than infection, although we believe that this risk is low as we selected patients on the basis of a clinical syndrome in combination with radiologic and microbiological criteria.

In conclusion, the proportion of pneumonia attributable to pneumococcus was high in adults in an urban referral hospital in Mozambique. All pneumococcal strains turned out to be penicillin susceptible as based on benzyl penicillin MIC results and there seems to be no need to abandon penicillin as the main treatment for pneumococcal pneumonia. At the same time, given the presence of strains with MICs > 0.5 mg/L, current standard penicillin dosing for pneumonia may be insufficient. For Mozambique, as well as for other sub-Saharan African countries, we would like to emphasize the need for the use of a standard dosage that would be effective against strains with penicillin MICs within the locally observed range. More extensive, continued monitoring is needed to guide prudent empiric antibiotic choices.

## Abbreviations

CLSI: Clinical and laboratory standards institute; CTX: Co-trimoxazole; CXR: Chest x-ray; EUCAST: European committee on antimicrobial susceptibility testing; HCB: Hospital Central da Beira; IU: International units; MIC: Minimal inhibitory concentration; RS: Respiratory syndrome; ICT: Immunochromatographic test; ND: Not done.

## Competing interests

The authors declare that they have no competing interests.

## Authors’ contributions

JCB, CS and JMP conceived of the study and designed it. JCB, SB, GM, ESG, EC, CM and JMP designed the study protocol. SB, GM and JCB carried out the study participant recruitment and screening. SB performed all microbiological analyses, under supervision of EC, JCB and CS. RS reviewed all CXRs. JCB and SB analysed the data and JCB, CS and JMP carried out the interpretation of the data. JCB drafted the manuscript. CS, RS and JMP critically revised the manuscript for intellectual content. All authors read and approved the final manuscript.

## Authors’ information

JCB and JMP are internists and infectious diseases specialists who have been collaborating extensively with the Faculty of Health Sciences of the Catholic University of Mozambique (UCM) in Beira and the Ministry of Health of the Republic of Mozambique (MISAU) since 2006 for the improvement and expansion of graduate and post-graduate internal medicine training, including infectious diseases.

## References

[B1] File TM Community-acquired pneumonia Lancet 2003 362 1991 2001 10.1016/S0140-6736(03)15021-0 14683661 PMC7119317

[B2] Hirschtick RE Glassroth J Jordan MC Wilcosky TC Wallace JM Kvale PA Markowitz N Rosen MJ Mangura BT Hopewell PC Pulmonary Complications of HIV Infection Study Group Bacterial pneumonia in persons infected with the human immunodeficiency virus N Engl J Med 1995 333 845 851 10.1056/NEJM199509283331305 7651475

[B3] Jordano Q Falcó V Almirante B Planes AM Del Valle O Ribera E Len O Pigrau C Pahissa A Invasive pneumococcal disease in patients infected with HIV: still a threat in the era of highly active antiretroviral therapy Clin Infect Dis 2004 38 1623 1628 10.1086/420933 15156452

[B4] Jones RN Jacobs MR Sader HS Evolving trends in *Streptococcus pneumoniae* resistance: implications for therapy of community-acquired bacterial pneumonia Int J Antimicrob Agents 2010 36 197 204 10.1016/j.ijantimicag.2010.04.013 20558045

[B5] Van Bambeke F Reinert RR Appelbaum PC Tulkens PM Peetermans WE Multidrug-resistant *Streptococcus pneumoniae* infections: current and future therapeutic options Drugs 2007 67 2355 2382 10.2165/00003495-200767160-00005 17983256

[B6] Vlieghe E Phoba MF Tamfun JJ Jacobs J Antibiotic resistance among bacterial pathogens in Central Africa: a review of the published literature between 1955 and 2008 Int J Antimicrob Agents 2009 34 295 303 10.1016/j.ijantimicag.2009.04.015 19540725

[B7] National Institute of Health (INS) of the Ministry of Health of the Republic of Mozambique (MISAU) Inquérito nacional de prevalência, riscos, comportamentais e informação sobre o HIV e SIDA em Moçambique. INSIDA 2009. Relatório preliminar sobre a prevalência da infecção por HIV [http://www.cncs.org.mz/index.php/por/HIV-SIDA-em-Mocambique/Mais-informacoes-sobre-HIV-SIDA-em-Mocambique]

[B8] European Committee on Antimicrobial Susceptibility Testing (EUCAST) Breakpoint tables for interpretation of MICs and zone diameters. Version 3.1 [http://www.eucast.org/clinical_breakpoints]

[B9] Scott JAG Hall AJ Muyodi C Lowe B Ross M Chohan B Mandaliya K Getambu E Gleeson F Drobniewski Aetiology, outcome and risk factors for mortality among adults with acute pneumonia in Kenya Lancet 2000 355 1225 1230 10.1016/S0140-6736(00)02089-4 10770305

[B10] Yoshimine H Oishi K Mubiru F Nalwoga H Takahashi H Amano H Ombasi P Watanabe K Joloba M Aisu T Ahmed K Shimada M Mugerwa R Nagatake T Community-acquired pneumonia in Ugandan adults: short-term parenteral ampicillin therapy for bacterial pneumonia Am J Trop Med Hyg 2001 64 172 177 11442214 10.4269/ajtmh.2001.64.172

[B11] Jokinen J Scott JAG Estimating the proportion of pneumonia attributable to pneumococcus in Kenyan adults. Latent class analysis Epidemiology 2010 21 719 725 10.1097/EDE.0b013e3181e4c4d5 20562627 PMC2923075

[B12] Weinstein MP Klugman KP Jones RN Rationale for revised penicillin susceptibility breakpoints versus *Streptococcus pneumoniae*: coping with antimicrobial susceptibility in an era of resistance Clin Infect Dis 2009 48 1596 1600 10.1086/598975 19400744

[B13] Comissão Técnica de Terapêutica e Farmácia Antibióticos. Formulário Nacional de Medicamentos, 5^a^ edição 2007 Maputo: Ministério da Saúde da República de Moçambique Volume 8 155

[B14] Reddy EA Shaw AV Crump JA Community-acquired bloodstream infections in Africa: a systematic review and meta-analysis Lancet Infect Dis 2010 10 417 432 10.1016/S1473-3099(10)70072-4 20510282 PMC3168734

[B15] Schito GC Felmingham D Susceptibility of *Streptococcus pneumoniae* to penicillin, azithromycin and telithromycin (PROTEKT 1999–2003) Int J Antimicrob Agents 2005 26 479 485 10.1016/j.ijantimicag.2005.04.022 16289710

[B16] Everett DB Mukaka M Denis B Gordon SB Carrol ED Van Oosterhout JJ Molyneux EM Molyneux M French N Heyderman RS Ten years of surveillance for invasive *Streptococcus pneumoniae* during the era of antiretroviral scale-up and cotrimoxazole prophylaxis in Malawi PLoS One 2011 6 e17765 10.1371/journal.pone.0017765 21423577 PMC3058053

[B17] Koornhof HJ Wasas A Klugman K Antimicrobial resistance in *Streptococcus pneumoniae*: a South African perspective Clin Infect Dis 1992 15 84 94 10.1093/clinids/15.1.84 1617077

